# Social Determinants of Health Screening Tools for Adults in Primary Care: Protocol for a Scoping Review

**DOI:** 10.2196/68668

**Published:** 2025-02-19

**Authors:** Julia Martínez-Alfonso, Fernando Sebastian-Valles, Vicente Martinez-Vizcaino, Nuria Jimenez-Olivas, Antonio Cabrera-Majada, Iván De los Mozos-Hernando, Shkelzen Cekrezi, Héctor Martínez-Martínez, Arthur Eumann Mesas

**Affiliations:** 1 Centro de Salud Daroca Departamento de Medicina Familiar y Comunitaria Salud Madrid Madrid Spain; 2 Departamento de Endocrinología y Nutrición, Hospital Universitario de La Princesa Instituto de Investigación Sanitaria de La Princesa Universidad Autónoma de Madrid Madrid Spain; 3 Centro de estudios sociosanitarios Universidad de Castilla-La Mancha Cuenca Spain; 4 Facultad de Ciencias de la Salud Universidad Autónoma de Chile Talca Chile

**Keywords:** social deprivation, social determinants of health, primary health care, social inequality, screening

## Abstract

**Background:**

Social determinants of health (SDH) have been shown to be predictors of health outcomes. Integrating SDH screening tools into primary care may help identify individuals or groups with a greater burden of social vulnerability and promote health equity.

**Objective:**

This study aimed (1) to identify the existing screening tools to assess social deprivation in adults in primary care settings; (2) to describe the characteristics of these tools and, where appropriate, their psychometric properties; (3) to describe their validity and reliability in those scales in which validation processes have been conducted; and (4) to identify evidence gaps and provide recommendations for future research.

**Methods:**

This study protocol was structured according to the Joanna Briggs Institute methodology for scoping reviews and reported according to the PRISMA-ScR (Preferred Reporting Items for Systematic Reviews and Meta-Analyses Extension for Scoping Reviews) guidelines. Furthermore, since not all SDH assessment tools are published as scientific papers, we will use a slightly modified form of the scoping review framework to retrieve specific information about specific tools for screening SDH in primary care contexts. The following electronic databases will be searched by 2 reviewers: MEDLINE (via PubMed), CINAHL Plus, Web of Science, and Scopus. In addition, the following sources will also be searched for gray literature: DART-Europe E-thesis Portal, OpenGrey, and Google Scholar. After the revision of inclusion and exclusion criteria, the titles, abstracts, and full text of the included studies will be separately screened by 2 reviewers. A PRISMA-ScR flowchart will be used to depict the sources of evidence screened, and data charting will be used to gain in-depth knowledge. The findings of the scoping review will be presented in both narrative and tabular formats, summarizing the existing literature on tools used for SDH in primary care settings. A critical analysis will be undertaken to address the variability in tool validation, cultural adaptability, and integration into different health care systems. Finally, key gaps in the existing evidence will be explored, and research priorities will be proposed, emphasizing the need for screening tools that are culturally sensitive, scalable, and easily integrated into primary care workflows. This critically appraised information may be useful for implementing SDH screening tools in primary care settings and may contribute to future research addressing feasibility and validation studies in different primary health care systems.

**Results:**

The study began in July 2024. Data collection is expected to be completed in April 2025, with publication expected in October 2025.

**Conclusions:**

This scoping review will provide a comprehensive and critical description of the available tools aimed at screening SDH in primary care settings. Incorporating these tools into routine care has been recognized as a key strategy for addressing health inequalities, given the growing evidence base on the influence of SDH on health outcomes.

**International Registered Report Identifier (IRRID):**

PRR1-10.2196/68668

## Introduction

### Background

According to the World Health Organization (WHO), social determinants of health (SDH) are the conditions in which people are born, grow, work, live, and age, along with the broader set of forces and systems that shape the conditions of daily life [1]. These determinants include a wide range of factors, such as income, education, employment, working conditions, housing, neighborhood environments, race, and gender [2]. On the other hand, health inequalities are defined as the systematic, avoidable, and unfair differences in health outcomes that can be observed between different groups of people, which are determined by the SDH [3].

In recent years, there has been a growing awareness of the significant impact of SDH on individual and population health outcomes. This has led to a transformation in health care practices and policies with greater recognition of the role of SDH in perpetuating health inequities and in providing a comprehensive understanding of a patient’s health [4].

SDH have been shown to be the predictors of health outcomes, including hospital readmissions [5], emergency department visits [6], multimorbidity burden [7], depression prognosis [8], and lower adherence to preventive measures [9,10]. Identifying individuals or groups with a greater burden of social vulnerability or with the greatest disparities in a particular disease can guide future actions to promote health equity [11], implement tailored social interventions, and direct future research [12]. However, despite the clear evidence of the importance of SDH and the need to address its root causes, there are several issues to be considered. First, without multidisciplinary engagement and workflows, along with the availability of social resources for subsequent referral [13], expectations may be raised without solutions being provided, leading to a loss of patient trust. Second, without intersectoral collaboration, long-term strategies, upstream proposals, and public health policies and workflows, we could fall into perpetuating the “fantasy paradigm” [14]—a parallel fantasy world in which proximal, downstream, and easily tackled exposures are posited as potential solutions to health inequalities [15].

Primary care settings are ideal for addressing SDH because they are often the first, and sometimes only, point of contact for patients within the health care system. They are also the place for multiple consultations with a significant social burden, where longitudinal continuity of care is provided and where clinicians are aware of the community health resources [16]. The importance of SDH screening in primary care is underscored by the fact that social needs are often unrecognized in clinical settings, leading to suboptimal care and poorer health outcomes [17]. Therefore, integrating SDH screening tools into primary care is not only consistent with the principles of holistic and patient-centered care but also represents a crucial step toward addressing the root causes of health disparities [18-20].

There is considerable variability in the implementation of SDH screening tools in primary care settings [21]. The absence of standardized screening tools and protocols, along with varying levels of knowledge and training among providers, hinders the ability to identify SDH-related needs and intervene appropriately [22]. Furthermore, the diverse nature of SDH, which covers a wide range of domains and is influenced by individual-, community-, and policy-level factors, poses a significant challenge to the development of comprehensive screening tools [23]. These tools must be sensitive enough to capture the complexity of social determinants while also being practical for use in time-constrained primary care settings. A number of SDH screening tools have been developed and implemented with varying degrees of success. These tools range from brief questionnaires integrated into electronic health records to more extensive assessments conducted through patient interviews [14,21,24]. The development of these tools is often context specific, taking into account the patient population, health care setting, and available resources for follow-up interventions [25]. However, the variability in the content, format, and application of these tools across different health care systems underscores the need for a comprehensive synthesis of available tools aimed at assessing their effectiveness, validity, and feasibility [14].

Primary care providers face numerous barriers to implementing SDH screening, including time constraints, lack of training, and uncertainty about how to address identified needs [17,26]. There are also concerns that screening may reveal problems that providers are ill-equipped to deal with, leading to increased stress and workload without a clear pathway for patient referral and intervention [14]. Without collaboration between sectors such as social work or community resources in SDH screening and subsequent referral, primary care clinicians alone may not be able to cope [26]. Therefore, an important aspect of evaluating SDH screening tools is to consider not only their ability to identify social determinants but also their integration into care processes, the availability of resources to address identified needs, and their impact on patient outcomes [27].

### Rationale for a Scoping Review

To avoid duplication of effort, a preliminary search conducted in July 2024 revealed that although several approaches to synthesizing the literature on the use of tools to screen for SDH in clinical settings had been published [17,21,28] or were in progress (Parry et al [29]), many of the known tools had not been published in scientific papers and could only be found on institutional websites or through references to their use in the scientific literature (eg, EPICES score [30]) or book chapters. Therefore, several approaches to synthesizing the existing literature were considered, and scoping was found to be the most appropriate for the needs of this study according to the reasons for deciding to undertake a scoping review proposed by Arksey and O’Malley [31], namely that the use of SDH screening tools in primary care is an emerging issue, and different tools have been proposed because the generalizability across countries and primary care systems may not be possible, so mapping these tools is of scientific interest. In addition, a standard systematic review would leave out numerous tools that have not been published in traditional databases of the scientific literature, such as websites of health institutions, books, and so on. Therefore, suggesting lines of research and methodologies can be of scientific interest.

### Study Aims

Our overall aim is to explore the literature describing the usefulness of SDH screening tools for adults in primary care settings. To achieve this, we will address the following specific objectives: (1) to identify existing screening tools for assessing social deprivation in adults in primary care settings; (2) to describe the characteristics of these tools, such as country, year of publication, and items included; (3) to describe their validity and reliability in those scales that have undergone validation processes; and (4) to identify evidence gaps and provide recommendations for future research.

## Methods

### Overview

In reviewing the existing literature, several approaches were considered, and a scoping review was found to be the most appropriate for the requirements of this study. Therefore, this study protocol was structured according to the 5-stage framework by Arksey and O’Malley [31]. In addition, the scoping review will be conducted according to the Joanna Briggs Institute methodology for scoping reviews [32] and reported following the PRISMA-ScR (Preferred Reporting Items for Systematic Reviews and Meta-Analyses Extension for Scoping Reviews) guidelines [33]. Furthermore, because not all the SDH assessment tools are published as scientific papers, we will use a slightly modified form of the scoping review framework outlined by Peters et al [34] to retrieve specific information about specific social deprivation screening tools in primary care contexts.

### Identifying the Research Question

Some differences can be observed between these two approaches [33,34]. In essence, both approaches can be complementary, as the former is a checklist for reporting a scoping review compatible with the Population, Concept, and Context framework that we have chosen to describe the research question of our review (Multimedia Appendix 1). Our population will consist of adults (aged 18 years or older); our concept of SDH tools is understood as the conditions in which people are born, grow, work, live, and age, and the broader set of forces and systems that shape the conditions of daily life [1]; and as a contextual framework, the tools should be applicable to primary care settings.

### Identifying Relevant Studies

The search strategy has been developed in collaboration with the research team and the participant librarians, adapting the PRISMA-ScR guidelines for literature search [35] to a scoping review. Two reviewers (JM-A and VM-V) independently searched the following electronic databases: MEDLINE (via PubMed), CINAHL Plus, Web of Science, and Scopus. The search strategy included terms related to the following descriptors, combined using Boolean operators: (1) SDH, (2) measurement tools, (3) validation studies, and (4) primary health care. All retrieved records will be imported into Mendeley (Elsevier) software, and duplicates will be removed. Textbox 1 provides the search strategy for the MEDLINE database as an example.

**Search strategy in MEDLINE.**

**Concept**

- (tool [Title/Abstract]) OR (“questionnaire”[Title/Abstract])) OR (“scale”[Title/Abstract])) OR (measurement[Title/Abstract])) OR (“test”[Title/Abstract])) OR (“measure”[Title/Abstract])) OR (“assessment”[Title/Abstract])) OR (“index”[Title/Abstract])) OR (“indexes”[Title/Abstract]) OR (“score”[Title/Abstract]) OR (screening[Title])

AND

**Social determinants of health**

- (poverty[Title/Abstract]) OR (poverty[MeSH Terms])) OR (socioeconomic status[Title/Abstract])) OR (low socioeconomic status[Title/Abstract])) OR (low socioeconomic status[MeSH Terms])) OR (Social Deprivation[MeSH Terms])) OR (Social Deprivation[Title/Abstract])) OR (Social Vulnerability[Title/Abstract])) OR (Social Vulnerability[MeSH Terms]) ) OR (Social Determinants of Health[Title/Abstract])) OR (Social Determinants of Health[MeSH Terms])) OR (“social class”[MeSH Terms])) OR (social determinants[Title/Abstract])) OR (socioeconomic factors[MeSH Terms])) OR (socioeconomic factors[Title/Abstract])) OR (deprivation[Title/Abstract]) OR (drivers [Title]) OR (Social Drivers of Health [Title]) OR (social needs[Title])

AND

**Validity**

- (validity [Title/Abstract]) OR (Feasibility Studies [MeSH Terms] OR (Feasibility[Title/Abstract])) OR (applicability[Title/Abstract])) OR (screening[Title/Abstract])) OR (validation[Title/Abstract])) OR (“validation studies as topic”[MeSH Terms])) OR (health outcome predictor[Title])

AND

**Context**

- (Primary Health Care [Title/Abstract]) OR (Primary Health Care[MeSH Terms])) OR (primary care[Title/Abstract])) OR (Family Practice[Title/Abstract])) OR (Family Practice[MeSH Terms])) OR (general practice[MeSH Terms])) OR (general practice[Title/Abstract])) OR (clinical setting[Title]) OR (outpatient [Title]) OR (ambulatory [Title]) OR (Internal Medicine[Title])

The search strategy and index terms will be adapted for each electronic database or information source.

In addition, several approaches will be used to search the gray literature, understood as materials and research produced by organizations outside of traditional academic publishing. First, we will search the DART-Europe E-thesis Portal for access to dissertations and OpenGrey, a system for information on gray literature in Europe. We will also search using keywords in Google Scholar, and the first 200 results sorted by relevance will be screened for suitability according to the recommendations of Haddaway et al [36]. Furthermore, a supplementary search of the reference lists of studies selected for inclusion in the review will be conducted to identify additional relevant studies. This will be followed by a systematic citation search using CitationChaser [37] to compile studies citing the papers selected for inclusion. If further information is required regarding the studies of key papers, the authors will be contacted accordingly. Finally, an iterative approach will be used to search the websites of health institutions in the main countries that have implemented health policies related to social inequalities in health. Mendeley will be used as reference manager software.

### Study Selection

The identified studies will be transferred to the web-based version of the Rayyan Systematic Review Tool [38] for further processing. Rayyan is a web-based tool designed to facilitate the screening process, which is a critical component of any systematic review. As recommended, two authors (JM-A and VM-V), after agreeing on a framework for screening papers according to the research objectives [33], will independently carry out the title and abstract selection of studies. Full papers will be obtained if the literature meets the inclusion criteria or if a decision cannot be made from the title or abstract alone. The review authors will resolve disagreements by consensus-based decision and, if necessary, by discussion within the group. In addition, we will record the specific reasons for the exclusion of each study.

For our scoping review, the inclusion criteria are given in Table 1. However, we will be aware that a reflexive process of the inclusion and exclusion criteria will be undertaken during the screening process, which will serve to consolidate the criteria [34].

**Table 1:** Inclusion and exclusion criteria based on the Population, Concept, and Context framework.

Scope parameters	Inclusion criteria	Exclusion criteria
Population	Adult participants (including older adults)	Pediatric population and adolescents
Concept	Screening tools for SDH^a^ that include more than one dimension of SDH and screening tools with social deprivation indexes	Screening tools for only one dimension of SDH (eg, screening tools for only food insecurity)
Context	Primary care settings	Other specialty or emergent care setting
Types of evidence	Full-text papers of empirical research studies (eg, validation studies, randomized controlled trials, and observational studies), study protocols, full-text conference proceedings, papers written in English, documents retrieved from institutional websites, and PhD dissertations	Reviews^b^, abstracts, posters, and papers for which we cannot obtain the full text or which are not written in English

^a^SDH: social determinants of health.

^b^Although systematic reviews will not be selected for inclusion, the studies included in these reviews will be reviewed to evaluate their possible inclusion in the scoping review.

### Critical Appraisal

As scoping reviews are primarily aimed at identifying and exploring the existing literature on a topic, it has been stated that a quality assessment is not applicable [34]. In our case, as the methods used to develop the SDH screening tool may not always be standardized, conducting a quality assessment is likely to be unsuccessful. However, a critical review assessing their ability to be incorporated into care processes, their relationship to the availability of resources to address identified needs, and their impact on patient outcomes will be included in the *Results* and *Discussion* sections.

### Charting the Data

A PRISMA-ScR [33] flowchart will be used to depict the sources of evidence screened, the assessment of documents’ eligibility (which may include tools extracted from institutional documents, not just papers), and the tools included in the review, along with reasons for exclusions at each stage.

The data charting is specific to scoping reviews and differs from the data extraction processes commonly used in other types of research synthesis designs, where data extraction is a more structured process often including statistical procedures. In contrast, data charting in scoping reviews is a more comprehensive approach that incorporates narrative information to describe details about how, why, and where the study was conducted [39]. Accordingly, a consensus-based, data-charting form will be used by the 2 reviewers, who will independently extract the data, discuss the results, and iteratively update the data-charting form. This data-charting table will include descriptive variables (year of publication, study design, setting, target population, and data source) and information about the aims and structure (dimensions, items, and procedures for completing the questions) of the tool and setting characteristics.

### Ethical Considerations

Ethical approval is not required for this review as it involves the analysis of publicly available empirical studies and the production of secondary data.

### Expected Outcomes

Following the publication of this protocol, the search for SDH screening tools, removal of duplicates, and study selection will take place (estimated time: 2 months). The findings of this scoping review will be presented in both narrative and tabular formats, summarizing the existing literature on tools used for SDH in primary care settings. The narrative summary will describe the scope and nature of the identified screening tools including their structure (domains, number of items, and how the information should be obtained [eg, questionnaires and digital platforms]) and the contexts in which they are applied. The integration of these tools into clinical workflows, as well as any evidence of their effectiveness in improving patient outcomes, will also be considered.

### Critical Analysis

#### Overview

The results will include a critical analysis of the strengths, limitations, and usability in primary care settings of the screening tools. This analysis will address the variability in tool validation, cultural adaptability, and integration into diverse health care systems. Potential biases or limitations in the implementation or outcomes of the tools, such as insufficient training of health care providers or limited follow-up on identified needs, will also be concerns to be discussed.

#### Research Gaps and Priorities

Key gaps in the existing evidence, such as social determinants not included in the tools, concerns about age or gender underrepresentation, and limited follow-up analysis of the usefulness of SDH screening on health outcomes, will also be critically examined. Finally, based on these findings, research priorities will be proposed, emphasizing the need for screening tools that are culturally sensitive, scalable, and easily integrated into primary care workflows (estimated time to complete these tasks: 6 months).

#### Dissemination of the Results

To ensure that the insights resulting from this review reach a variety of stakeholders, such as health care practitioners, policy makers, and academics, the findings will be disseminated using several dissemination strategies including reporting results in open-access journals and scientific conferences. In addition, stakeholders will be engaged at every stage of the review process to facilitate the adoption and implementation of evidence-based screening tools in clinical settings, thereby increasing their impact on health equity (estimated time: 6 months).

## Results

The study began in July 2024. Data collection is expected to be completed in April 2025, with publication expected in October 2025.

## Discussion

### Principal Findings

This scoping review will provide a comprehensive and critical description of the available tools aimed at screening SDH in primary care settings. Incorporating these tools into routine care has been recognized as a key strategy for addressing health inequalities, given the growing evidence on the influence of SDH on health outcomes. Nonetheless, the absence of a comprehensive review makes it difficult for health care practitioners to select the most appropriate tools for their context and patient populations.

The importance of identifying and addressing the social needs of the patients attending health care settings has been highlighted by Gottlieb and Fichtenberg [40], and some scientific contributions have previously addressed some issues related to the implementation of SDH screening tools in clinical settings, such as the coverage and economic evaluation of these tools [41], the evaluation of interventions linking social and medical care [42], the barriers to the implementation of SDH in electronic health records [43], and its integration in nonspecific clinical settings [44]. In addition, a report from the US Preventive Services Task Force alerted on the importance of considering SDH in the recommendations of preventive interventions in primary care [19]. However, a catalog of currently available screening tools, their scope, structure, and dimensions is lacking. Furthermore, this review will assess the contextual elements such as resource accessibility and stakeholder involvement (patients, practitioners, and health care providers) that influence the implementation and effectiveness of these technologies in primary care. This review will map the body of literature to identify potential gaps and areas for additional research, including tool validation in varied populations, tool influence on clinical outcomes, and tool integration into larger care systems.

Including SDH screening tools for children in this review might seem to make our review more coherent, but it would greatly increase the complexity of the review. The tools for children and adults are very different. Overall, measuring SDH in children should emphasize developmental needs, relationships with caregivers, and early life conditions, whereas measures for adults tend to focus more on employment, social deprivation, and cumulative social conditions, as well as focus on the individual rather than indirectly asking the caregiver about the child’s health [45]. Therefore, a review of SDH in the pediatric population requires a synthesis study, probably with a different methodological approach, focusing exclusively on this topic.

A key strength of this protocol lies in its systematic approach, which adheres to the current methodological frameworks for scoping reviews. This ensures that the review process will be transparent and reproducible, while allowing a broad range of study designs and inclusion settings. However, a limitation of this review is the potential for missing unpublished or non–English-language studies, which may result in an incomplete understanding of the global landscape of SDH screening tools.

**Conclusions**

This scoping review will provide a valuable synthesis of the available SDH screening tools applicable in primary care and identify critical gaps in the existing literature. This review will provide insights into the implementation of these tools, while identifying areas for further research, including validation in diverse populations. Ultimately, this work aims to support the integration of SDH screening into routine care, contributing to efforts to reduce health inequalities and improve patient outcomes.

## Appendix

Multimedia Appendix 1 PRISMA-P (Preferred Reporting Items for Systematic Review and Meta-Analysis Protocols) checklist.

## Abbreviations

PRISMA-ScR: Preferred Reporting Items for Systematic Reviews and Meta-Analyses Extension for Scoping Reviews
SDH: social determinants of health
WHO: World Health Organization
